# Supplementary figures and images for: “Difficult to Sedate”: Successful Implementation of a Benzodiazepine-Sparing Analgosedation-Protocol in Mechanically Ventilated Children
Source: Children (Basel). 2021 Apr 28;8(5):348. doi: 10.3390/children8050348 (PMC8146538; doi:10.3390/children8050348)

Supplemental Figure S1: PICU: Analgo-Sedation Protocol

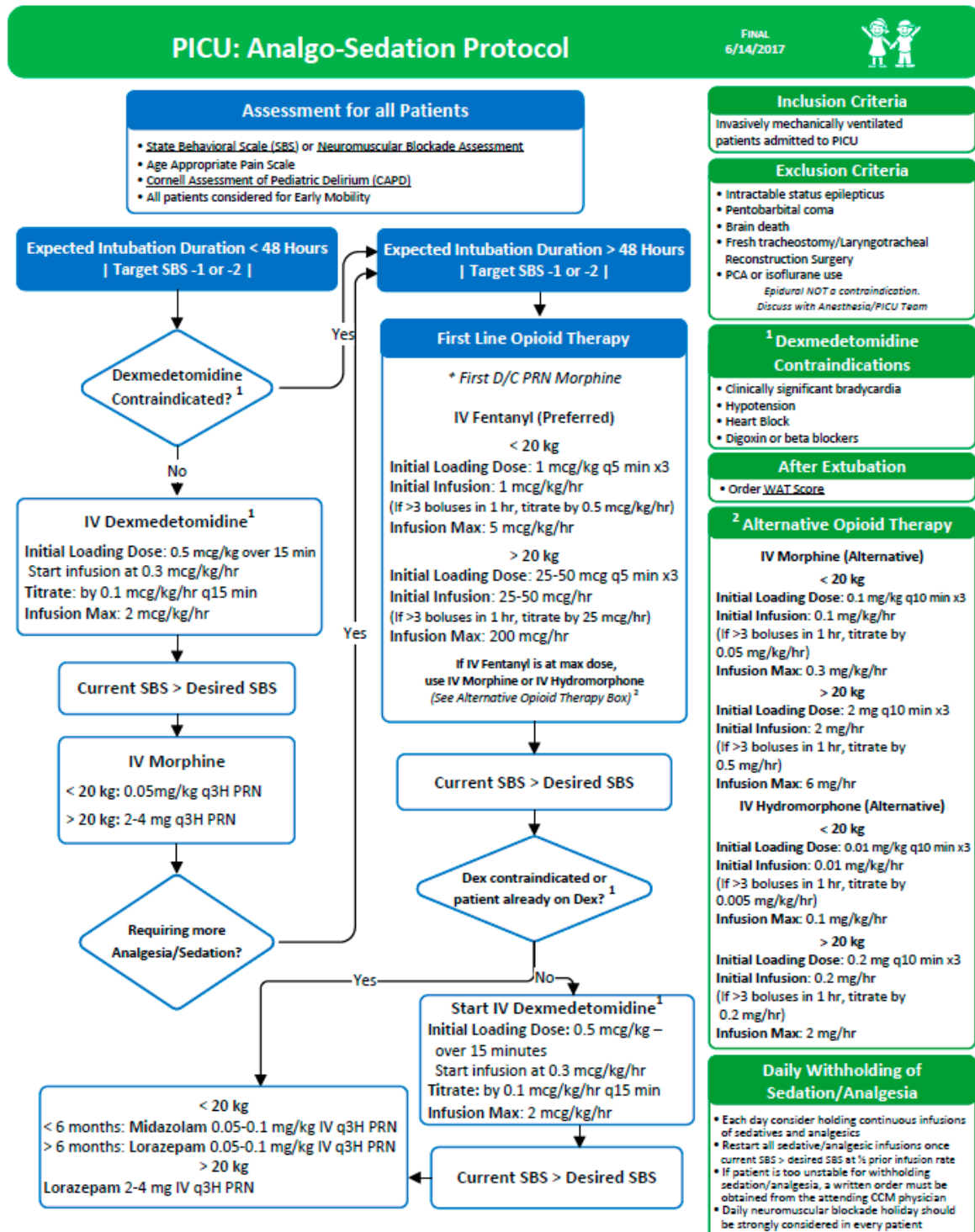

Supplement: Supplementary file 1 [file children-08-00348-s001.zip › children-1167961-supplementary.pdf]
